# Associations of the Triglyceride-Glucose Index With the Risk of Hypertension and Prehypertension

**DOI:** 10.31083/RCM47640

**Published:** 2026-10-14

**Authors:** Yue Jiang, Qi Qi, Xinyu Wu, Lei Li, Chunhui Yin, Cangtuo Li, Bocheng Yue, Quanle Han, Shouling Wu, Kangbo Li

**Affiliations:** ^1^Catheterization Unit, Tangshan Gongren Hospital, 063000 Tangshan, Hebei, China; ^2^Department of Cardiology, Tangshan Gongren Hospital, 063000 Tangshan, Hebei, China; ^3^Graduate School of Hebei Medical University, 050017 Shijiazhuang, Hebei, China; ^4^Department of Vascular and Interventional Radiology, Tangshan Gongren Hospital, 063000 Tangshan, Hebei, China; ^5^Department of Cardiology, Kailuan General Hospital, 063000 Tangshan, Hebei, China; ^6^School of Clinical Medicine, North China University of Science and Technology, 063210 Tangshan, Hebei, China

**Keywords:** triglyceride-glucose index, insulin resistance, hypertension, prehypertension

## Abstract

**Background:**

This study aimed to explore the associations between the triglyceride-glucose (TyG) index and the risk of developing hypertension and prehypertension in a Chinese population from North China.

**Methods:**

This study included two sub-studies. Sub-study 1 examined the association between the TyG index and the risk of hypertension. A total of 101,510 participants (81,110 men and 20,400 women, aged 18–98 years) were enrolled in the Kailuan study. Of these, 44,655 individuals with a history of hypertension were excluded, and 56,855 subjects were included in the analysis. Sub-study 2 aimed to examine the association between the TyG index and the risk of prehypertension. A total of 44,655 individuals with a history of hypertension and 35,777 individuals with a history of prehypertension were excluded, leaving 21,078 subjects for analysis. Participants were categorized into four groups (quartiles 1–4) based on the associated baseline TyG index. Cox proportional hazards regression and restricted cubic spline analyses were used to investigate the association between the TyG index and the risk of hypertension and prehypertension.

**Results:**

In sub-study 1, during a median follow-up of 13.94 years, 5825 incident cases of hypertension were reported. Compared with the first quartile, the fourth quartile had the highest hazard ratio (HR) for incident hypertension (HR: 1.352; 95% confidence interval (CI): 1.241–1.473). In sub-study 2, during a median follow-up of 3.97 years, 16,982 incident cases of prehypertension were reported. Compared with the first quartile, the fourth quartile had the highest HR for incident prehypertension (HR: 1.148; 95% CI: 1.092–1.207).

**Conclusions:**

These findings indicate that an elevated TyG index is associated with an increased risk of hypertension and prehypertension.

**Clinical Trial Registration:**

ChiCTR-TNRC-11001489, https://www.chictr.org.cn/showproj.html?proj=8050.

## 1. Introduction

In the year 2024, it was estimated that approximately 1.4 billion individuals aged between 30 and 79 years were affected by hypertension globally; however, less than 20% of them had their condition effectively managed [1]. A recent nationwide survey in China revealed that around 244.5 million adults were diagnosed with hypertension, resulting in an overall crude prevalence of 27.9% [2]. The global prevalence of hypertension is increasing due to the aging population and heightened exposure to lifestyle risk factors [3]. Recent research has suggested that insulin resistance (IR) may play a role in the development of hypertension [4]. Reduced sensitivity of tissues to insulin’s metabolic effects is a hallmark of various pathological states collectively known as “cardiometabolic syndrome” [5]. The triglyceride-glucose (TyG) index is a simple, readily available laboratory method for assessing IR. Nevertheless, the impact of TyG index levels on the risk of developing new-onset hypertension is not yet fully elucidated. Consequently, this study seeks to explore the associations between the TyG index and the risk of hypertension and prehypertension in a Chinese population of North China.

## 2. Methods

### 2.1 Study Population

The study population was sourced from the Kailuan study (registration number in the Chinese Clinical Trial Registry: ChiCTR-TNRC-11001489, https://www.chictr.org.cn/showproj.html?proj=8050). The Kailuan study is a prospective cohort investigation centered on the Kailuan community in Tangshan, a major industrial city in North China. Between June 2006 and October 2007, 155,418 employees aged 18 years and older (including retirees) from the Kailuan Group were invited to participate. A total of 101,510 participants (81,110 men and 20,400 women) aged 18–98 years were enrolled in the study [6]. Participants underwent in-person interviews, laboratory assessments, and physical examinations during the initial survey and at subsequent biennial visits since 2006. The present study includes two sub-studies: Sub-study 1 explored the association between the TyG index and the risk of hypertension. 44,655 individuals with a history of hypertension were excluded, and 56,855 subjects were analyzed. Sub-study 2 sought to explore the association between the TyG index and the risk of prehypertension. After excluding 44,655 individuals with a history of hypertension and 35,777 individuals with a history of prehypertension, 21,078 participants remained in the study (Fig. 1).

**Fig. 1.**
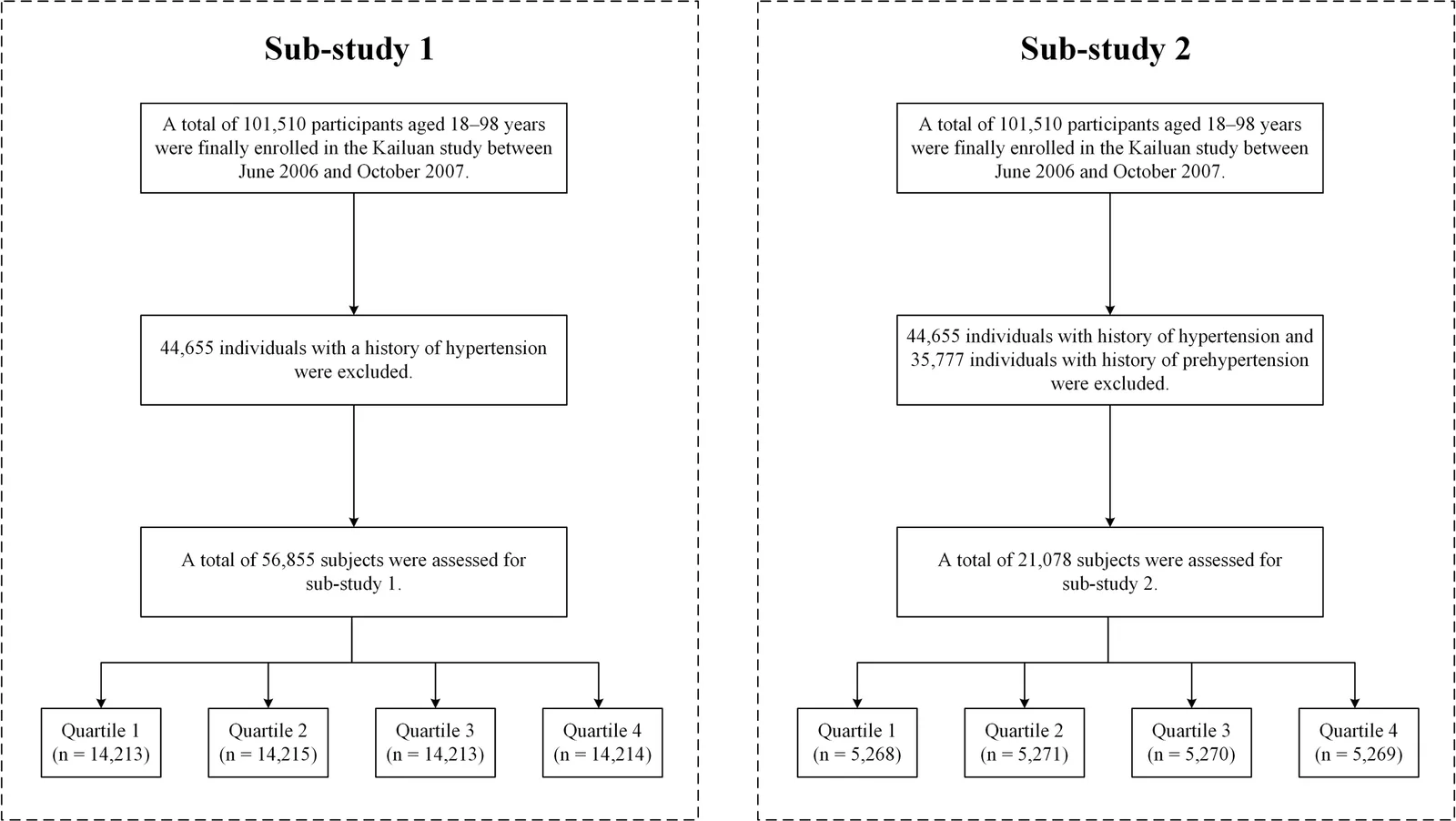
**Study flowchart**.

### 2.2 Calculation of the TyG Index

The TyG index was computed as Ln [triglycerides (TG) (mg/dL) × fasting blood glucose (FBG) (mg/dL)/2]. Participants were stratified into four groups according to quartiles of the TyG index levels: quartile 1: 4.37–8.07, quartile 2: 8.08–8.46, quartile 3: 8.47–8.91, quartile 4: 8.92–12.25 in sub-study 1. Quartile 1: 4.65–7.96, quartile 2: 7.97–8.34, quartile 3: 8.35–8.78, quartile 4: 8.79–11.94 in sub-study 2.

### 2.3 Diagnosis of Hypertension and Prehypertension

All participants were asked to rest for over 5 minutes and avoid consuming coffee or tea before having their blood pressure assessed by trained personnel using a mercury sphygmomanometer with a properly sized cuff, in accordance with established protocols. Three measurements of systolic blood pressure (SBP) and diastolic blood pressure (DBP) were taken at 5-minute intervals after participants had rested in a chair for at least 5 minutes. Blood pressure was measured three times; the average reading was used for the final analysis. Hypertension was defined as an SBP ≥140 mmHg or a DBP ≥90 mmHg, according to the World Health Organization guidelines for hypertension [7]. Prehypertension was defined as a SBP ranging from 120 to 139 mmHg and/or a DBP between 80 and 89 mmHg, occurring in the absence of antihypertensive medication [8].

### 2.4 Statistical Analysis

All analyses were conducted using SAS version 9.4 (SAS Institute, Inc., Cary, NC, USA). Continuous variables were presented as mean ± standard deviation for normally distributed data, and as median (25th percentile–75th percentile) for skewed data. Categorical variables were typically presented as counts or frequencies. The cumulative incidence of new-onset hypertension and prehypertension in each group was estimated using the Kaplan-Meier method, and the log-rank test was utilized to assess between-group differences. Cox proportional hazards regression analysis was used to investigate the associations between the TyG index and the risk of hypertension and prehypertension. The restricted cubic spline regression (RCS) analysis was used to assess a non-linear relationship between the TyG index and the risk of hypertension or prehypertension. Subgroup analyses were conducted based on age, sex, and body mass index (BMI). A Bonferroni-corrected *p* value threshold (0.05/3 ≈ 0.017) was applied to the results of interaction tests. Sensitivity analyses were performed by excluding participants who experienced hypertension during the first 2 years of follow-up, those who were diagnosed with diabetes during follow-up, individuals who experienced myocardial infarction and stroke, and those who died from any cause during the follow-up period.

## 3. Results

### 3.1 Baseline Characteristics

Sub-study 1 evaluated a total of 56,855 individuals at baseline, comprising 43,090 men and 13,765 women. Sub-study 2 evaluated a total of 21,078 individuals at baseline, comprising 14,266 men and 6812 women. The lowest age, lowest levels of BMI, FBG, total cholesterol, TG, low-density lipoprotein cholesterol, high sensitivity C-reactive protein, and blood pressure, and the lowest proportions of current smokers, and current drinkers, were observed in quartile 1 of two sub-studies (Tables 1,2).

**Table 1.** **Baseline characteristics**.

Variables	Total (n = 56,855)	Q1 (n = 14,213)	Q2 (n = 14,215)	Q3 (n = 14,213)	Q4 (n = 14,214)	*p* value
Age, years	48.84 ± 12.65	47.44 ± 13.54	48.67 ± 12.79	49.50 ± 12.36	49.77 ± 11.73	<0.001
Male, n (%)	43,090 (75.79)	9665 (68.00)	10,582 (74.44)	11,094 (78.06)	11,749 (82.66)	<0.001
BMI, kg/m^2^	24.31 ± 3.33	22.86 ± 3.05	23.89 ± 3.17	24.77 ± 3.16	25.63 ± 3.28	<0.001
SBP, mmHg	117.37 ± 11.06	114.90 ± 11.62	116.91 ± 11.08	117.90 ± 10.76	119.75 ± 10.19	<0.001
DBP, mmHg	76.33 ± 6.89	74.79 ± 7.31	76.06 ± 6.99	76.76 ± 6.59	77.69 ± 6.30	<0.001
TyG index	8.54 ± 0.67	7.76 ± 0.28	8.28 ± 0.11	8.68 ± 0.13	9.44 ± 0.46	<0.001
FBG, mmol/L	5.31 ± 1.50	4.75 ± 0.65	5.02 ± 0.72	5.27 ± 0.95	6.21 ± 2.45	<0.001
TC, mmol/L	4.88 ± 1.08	4.58 ± 0.90	4.84 ± 0.94	5.03 ± 0.97	5.06 ± 1.37	<0.001
TG, mmol/L	1.17 (0.82–1.75)	0.65 (0.54–0.75)	1.00 (0.89–1.11)	1.41 (1.25–1.61)	2.47 (1.98–3.44)	<0.001
HDL-C, mmol/L	1.53 ± 0.39	1.56 ± 0.40	1.54 ± 0.38	1.52 ± 0.38	1.49 ± 0.40	<0.001
LDL-C, mmol/L	2.30 ± 0.86	2.12 ± 0.88	2.33 ± 0.83	2.40 ± 0.81	2.35 ± 0.88	<0.001
hs-CRP, mg/L	0.73 (0.29–2.10)	0.60 (0.20–1.90)	0.65 (0.24–1.87)	0.79 (0.30–2.02)	0.99 (0.38–2.60)	<0.001
Diabetes, n (%)	2384 (4.19)	570 (4.01)	433 (3.05)	561 (3.95)	820 (5.77)	<0.001
Exercise regularly, n (%)	51,615 (90.78)	12,976 (91.30)	12,898 (90.74)	12,912 (90.85)	12,829 (90.26)	0.054
Current smoker, n (%)	20,331 (35.76)	4604 (32.39)	4885 (34.37)	5124 (36.05)	5718 (40.23)	<0.001
Current drinker, n (%)	22,154 (38.97)	5241 (36.87)	5303 (37.31)	5453 (38.37)	6157 (43.32)	<0.001
Snoring, n (%)	20,663 (36.34)	4891 (34.41)	4864 (34.22)	5125 (36.06)	5783 (40.69)	<0.001
Hypolipidemic drugs, n (%)	1186 (2.09)	57 (0.40)	151 (1.06)	247 (1.74)	731 (5.14)	<0.001
Hypoglycemic drugs, n (%)	2687 (4.73)	336 (2.36)	492 (3.46)	667 (4.69)	1192 (8.39)	<0.001

Abbreviations: BMI, body mass index; SBP, systolic blood pressure; DBP, diastolic blood pressure; TyG index, triglyceride-glucose index; FBG, fasting blood glucose; TC, total cholesterol; TG, triglycerides; HDL-C, high-density lipoprotein cholesterol; LDL-C, low-density lipoprotein cholesterol; hs-CRP, high sensitivity C-reactive protein.

**Table 2.** **Baseline characteristics**.

Variables	Total (n = 21,078)	Q1 (n = 5268)	Q2 (n = 5271)	Q3 (n = 5270)	Q4 (n = 5269)	*p* value
Age, years	46.53 ± 12.96	44.19 ± 13.53	45.98 ± 12.76	47.22 ± 12.81	48.71 ± 12.30	<0.001
Male, n (%)	14,266 (67.68)	3062 (58.12)	3389 (64.30)	3751 (71.18)	4064 (77.13)	<0.001
BMI, kg/m^2^	23.57 ± 3.22	22.24 ± 2.95	23.13 ± 3.07	23.89 ± 3.06	25.01 ± 3.13	<0.001
SBP, mmHg	106.98 ± 8.53	105.81 ± 8.63	106.70 ± 8.70	107.11 ± 8.28	108.31 ± 8.31	<0.001
DBP, mmHg	69.93 ± 5.97	69.25 ± 6.12	69.80 ± 5.98	70.07 ± 5.79	70.61 ± 5.88	<0.001
TyG index	8.40 ± 0.65	7.66 ± 0.28	8.16 ± 0.11	8.54 ± 0.12	9.26 ± 0.45	<0.001
FBG, mmol/L	5.18 ± 1.39	4.68 ± 0.62	4.93 ± 0.70	5.15 ± 0.86	5.96 ± 2.28	<0.001
TC, mmol/L	4.80 ± 1.01	4.49 ± 0.90	4.75 ± 0.91	4.92 ± 0.96	5.05 ± 1.16	<0.001
TG, mmol/L	1.05 (0.74–1.56)	0.60 (0.50–0.68)	0.89 (0.80–1.00)	1.26 (1.12–1.43)	2.13 (1.75–2.84)	<0.001
HDL-C, mmol/L	1.57 ± 0.37	1.55 ± 0.39	1.53 ± 0.37	1.50 ± 0.35	1.46 ± 0.37	<0.001
LDL-C, mmol/L	2.26 ± 0.82	2.05 ± 0.82	2.25 ± 0.81	2.37 ± 0.78	2.36 ± 0.83	<0.001
hs-CRP, mg/L	0.71 (0.30–2.16)	0.60 (0.20–2.00)	0.63 (0.23–1.83)	0.73 (0.30–2.05)	1.00 (0.40–2.75)	<0.001
Diabetes, n (%)	1031 (4.89)	30 (0.57)	74 (1.40)	150 (2.85)	777 (14.75)	<0.001
Exercise regularly, n (%)	19,024 (90.26)	4787 (90.87)	4768 (90.46)	4760 (90.32)	4709 (89.37)	0.125
Current smoker, n (%)	7544 (35.79)	1597 (30.32)	1783 (33.83)	1951 (37.02)	2213 (42.00)	<0.001
Current drinker, n (%)	8419 (39.94)	1882 (35.73)	2039 (38.68)	2126 (40.34)	2372 (45.02)	<0.001
Snoring, n (%)	8121 (38.53)	1836 (34.85)	1960 (37.18)	2000 (37.95)	2325 (44.13)	<0.001
Hypolipidemic drugs, n (%)	91 (0.43)	13 (0.25)	15 (0.28)	17 (0.32)	46 (0.87)	<0.001
Hypoglycemic drugs, n (%)	279 (1.32)	13 (0.25)	29 (0.55)	44 (0.83)	193 (3.66)	<0.001

### 3.2 The TyG Index and the Risk of Incident Hypertension and Prehypertension

In sub-study 1, during a median (interquartile range [IQR]) follow-up of 13.94 (13.54, 14.18) years, 5825 cases of incident hypertension were reported. The cumulative incidence of hypertension varies across quartiles: 7.30% in quartile 1, 9.67% in quartile 2, 11.55% in quartile 3, and 14.30% in quartile 4 (*p* < 0.001; Fig. 2A). When comparing the first quartile, the fourth quartile had a hazard ratio (HR) (95% confidence interval (CI) of 1.352 (1.241–1.473)) after adjustment for confounding factors (Table 3). Sensitivity analysis validated the robustness of the results (Table 4). The RCS regression analysis demonstrated a linear relationship between the TyG index and the risk of new-onset hypertension (*p* for overall < 0.001; *p* for non-linearity = 0.148) (Fig. 3A).

**Fig. 2.**
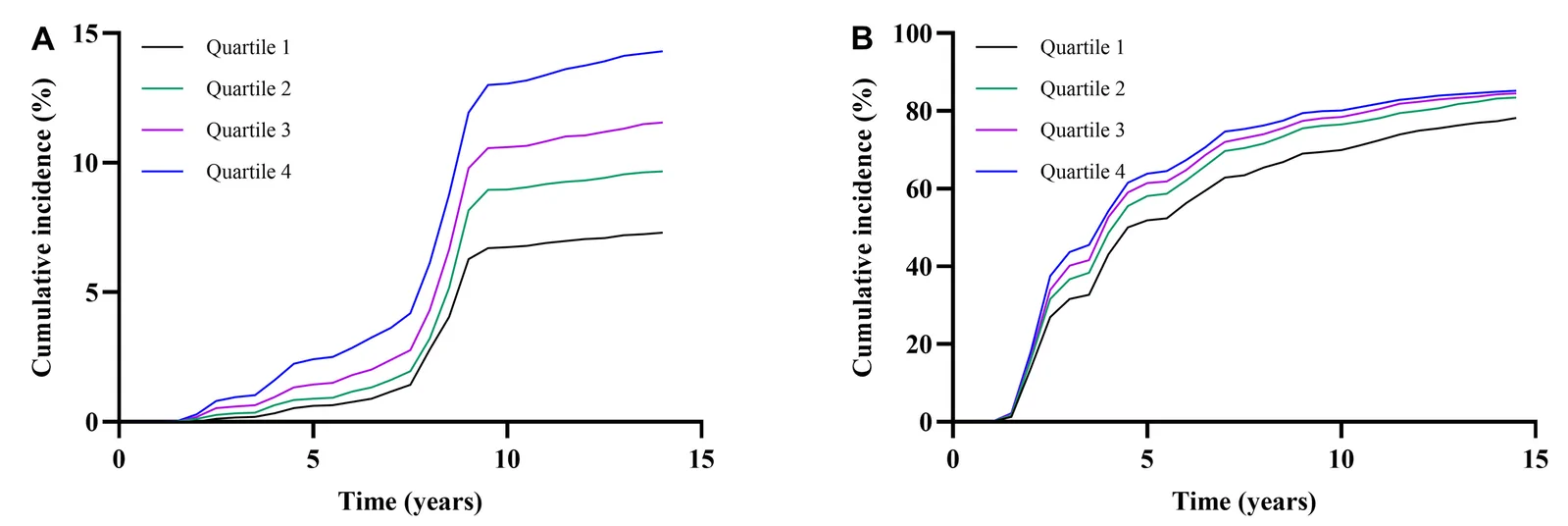
**The association between the TyG index and the cumulative incidence of (A) hypertension and (B) prehypertension**.

**Table 3.** **Hazard ratios for risk of hypertension according to TyG index quartiles**.

		β	SE	χ^2^	*p* value	HR	95% CI
Model 1							
	Q1	-	-	-	-	1.000	-
	Q2	0.293	0.042	48.921	<0.001	1.340	1.235–1.455
	Q3	0.473	0.040	136.609	<0.001	1.605	1.482–1.737
	Q4	0.699	0.039	321.439	<0.001	2.011	1.863–2.171
Model 2							
	Q1	-	-	-	-	1.000	-
	Q2	0.270	0.042	41.292	<0.001	1.309	1.206–1.422
	Q3	0.436	0.041	115.338	<0.001	1.546	1.428–1.675
	Q4	0.647	0.039	271.990	<0.001	1.909	1.768–2.062
Model 3							
	Q1	-	-	-	-	1.000	-
	Q2	0.143	0.043	10.805	0.001	1.153	1.059–1.256
	Q3	0.201	0.043	21.713	<0.001	1.222	1.123–1.330
	Q4	0.302	0.044	47.626	<0.001	1.352	1.241–1.473

Model 1 was unadjusted. Model 2 was adjusted for age and sex. Model 3 was adjusted for variables included in Model 2, and body mass index, systolic blood pressure, diastolic blood pressure, total cholesterol, high-density lipoprotein cholesterol, low-density lipoprotein cholesterol, high sensitivity C-reactive protein, diabetes history, physical activity, status of smoking, status of drinking, snoring intensity, hypolipidemic drugs usage, hypoglycemic drugs.

**Table 4.** **Sensitivity analysis for risk of hypertension according to the TyG index quartiles**.

		β	SE	χ^2^	*p* value	HR	95% CI
Analysis 1							
	Q1	-	-	-	-	1.000	-
	Q2	0.127	0.045	8.131	0.004	1.135	1.041–1.239
	Q3	0.178	0.044	16.153	<0.001	1.195	1.096–1.304
	Q4	0.256	0.045	31.972	<0.001	1.291	1.182–1.411
Analysis 2							
	Q1	-	-	-	-	1.000	-
	Q2	0.145	0.044	11.179	<0.001	1.157	1.062–1.260
	Q3	0.205	0.043	22.485	<0.001	1.227	1.128–1.336
	Q4	0.306	0.044	48.757	<0.001	1.359	1.247–1.481
Analysis 3							
	Q1	-	-	-	-	1.000	-
	Q2	0.148	0.044	11.569	<0.001	1.160	1.065–1.264
	Q3	0.203	0.043	21.986	<0.001	1.225	1.126–1.334
	Q4	0.308	0.044	49.179	<0.001	1.361	1.249–1.484
Analysis 4							
	Q1	-	-	-	-	1.000	-
	Q2	0.145	0.046	9.929	0.002	1.156	1.056–1.265
	Q3	0.231	0.045	26.041	<0.001	1.260	1.153–1.377
	Q4	0.301	0.046	42.301	<0.001	1.351	1.234–1.479

Model was adjusted for age, sex, body mass index, systolic blood pressure, diastolic blood pressure, total cholesterol, high-density lipoprotein cholesterol, low-density lipoprotein cholesterol, high sensitivity C-reactive protein, diabetes history, physical activity, status of smoking, status of drinking, snoring intensity, hypolipidemic drugs usage, hypoglycemic drugs usage. Analysis 1 excluded participants experienced hypertension during the first 2 years of follow-up. Analysis 2 excluded participants experienced diabetes during follow-up. Analysis 3 excluded participants experienced myocardial infarction and stroke during follow-up. Analysis 4 excluded all-cause death during follow-up.

**Fig. 3.**
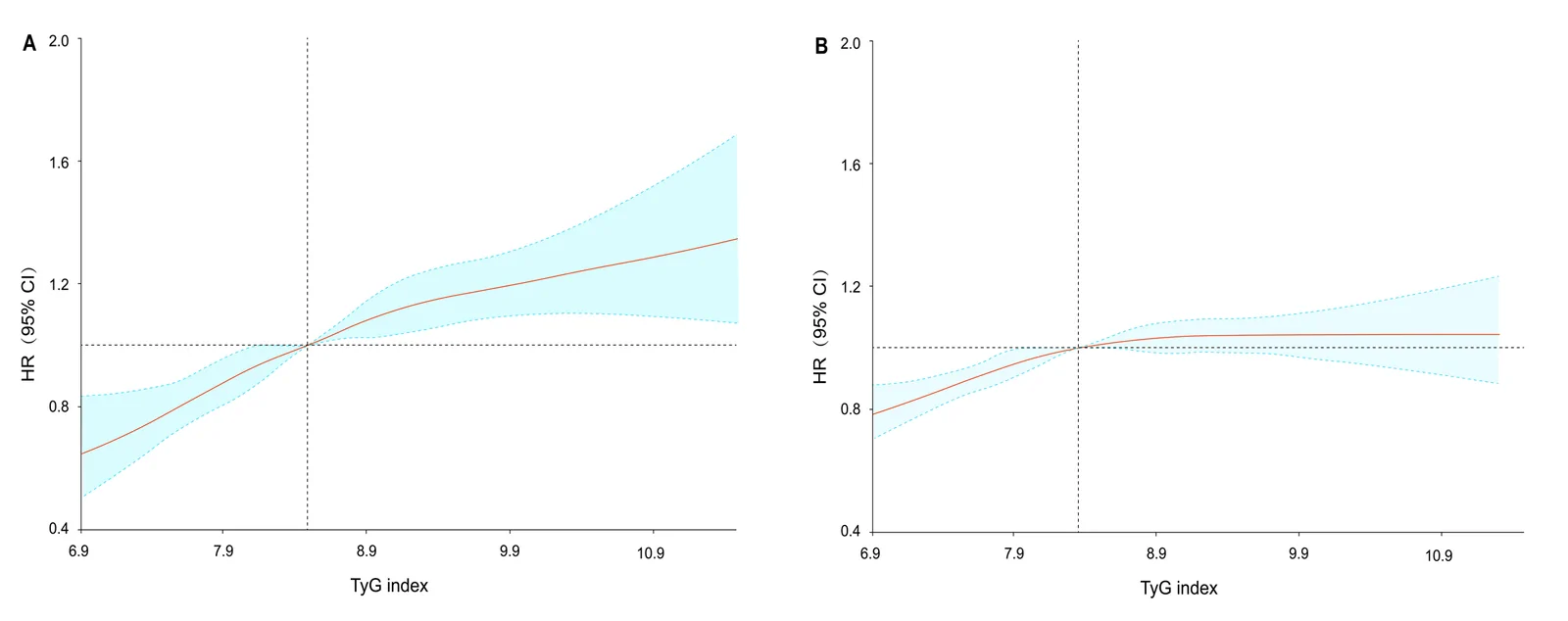
**Multivariable adjusted spline curve for association between TyG index and risk of (A) hypertension and (B) prehypertension**. Model was adjusted for age, sex, body mass index, systolic blood pressure, diastolic blood pressure, total cholesterol, high-density lipoprotein cholesterol, low-density lipoprotein cholesterol, high sensitivity C-reactive protein, diabetes history, physical activity, status of smoking, status of drinking, snoring intensity, hypolipidemic drugs usage, hypoglycemic drugs usage. Five knots were placed at the 5th, 25th, 50th, 75th, and 95th percentiles of the TyG index distribution.

In sub-study 2, during a median (IQR) follow-up of 3.97 (2.25, 8.54) years, 16,982 incident cases of prehypertension were reported. The cumulative incidence of prehypertension varies across quartiles: 78.24% in quartile 1, 83.45% in quartile 2, 84.51% in quartile 3, and 85.19% in quartile 4 (*p *< 0.001; Fig. 2B). When comparing the first quartile, the fourth quartile had a HR (95% CI) of 1.148 (1.092–1.207) after adjustment for confounding factors (Table 5). Sensitivity analysis validated the robustness of the results (Table 6). The RCS regression analysis revealed a non-linear relationship between the TyG index and the risk of new-onset prehypertension (*p* for overall < 0.001; *p* for non-linearity = 0.011) (Fig. 3B).

**Table 5.** **Hazard ratios for risk of prehypertension according to TyG index quartiles**.

		β	SE	χ^2^	*p* value	HR	95% CI
Model 1							
	Q1	-	-	-	-	1.000	-
	Q2	0.170	0.022	59.816	<0.001	1.185	1.135–1.238
	Q3	0.236	0.022	115.882	<0.001	1.266	1.213–1.322
	Q4	0.294	0.022	179.871	<0.001	1.342	1.285–1.401
Model 2							
	Q1	-	-	-	-	1.000	-
	Q2	0.162	0.022	54.143	<0.001	1.176	1.126–1.228
	Q3	0.219	0.022	97.501	<0.001	1.244	1.191–1.299
	Q4	0.268	0.022	143.449	<0.001	1.308	1.252–1.366
Model 3							
	Q1	-	-	-	-	1.000	-
	Q2	0.098	0.023	18.413	<0.001	1.103	1.055–1.154
	Q3	0.126	0.024	28.614	<0.001	1.135	1.083–1.188
	Q4	0.138	0.025	29.321	<0.001	1.148	1.092–1.207

Model 1 was unadjusted. Model 2 was adjusted for age and sex. Model 3 was adjusted for variables included in Model 2, and body mass index, systolic blood pressure, diastolic blood pressure, total cholesterol, high-density lipoprotein cholesterol, low-density lipoprotein cholesterol, high sensitivity C-reactive protein, diabetes history, physical activity, status of smoking, status of drinking, snoring intensity, hypolipidemic drugs usage, hypoglycemic drugs.

**Table 6.** **Sensitivity analysis for risk of prehypertension according to the TyG index quartiles**.

		β	SE	χ^2^	*p* value	HR	95% CI
Analysis 1							
	Q1	-	-	-	-	1.000	-
	Q2	0.126	0.035	12.591	<0.001	1.134	1.058–1.215
	Q3	0.115	0.037	9.477	0.002	1.122	1.043–1.208
	Q4	0.136	0.041	10.965	<0.001	1.146	1.057–1.242
Analysis 2							
	Q1	-	-	-	-	1.000	-
	Q2	0.105	0.023	21.056	<0.001	1.111	1.062–1.162
	Q3	0.145	0.024	37.684	<0.001	1.156	1.104–1.211
	Q4	0.165	0.026	41.771	<0.001	1.179	1.122–1.240
Analysis 3							
	Q1	-	-	-	-	1.000	-
	Q2	0.109	0.023	22.411	<0.001	1.115	1.066–1.166
	Q3	0.152	0.024	41.529	<0.001	1.165	1.112–1.220
	Q4	0.168	0.026	43.322	<0.001	1.183	1.125–1.244
Analysis 4							
	Q1	-	-	-	-	1.000	-
	Q2	0.110	0.024	21.650	<0.001	1.116	1.066–1.169
	Q3	0.155	0.024	40.422	<0.001	1.168	1.113–1.225
	Q4	0.167	0.026	40.097	<0.001	1.181	1.122–1.244

Model was adjusted for age, sex, body mass index, systolic blood pressure, diastolic blood pressure, total cholesterol, high-density lipoprotein cholesterol, low-density lipoprotein cholesterol, high sensitivity C-reactive protein, diabetes history, physical activity, status of smoking, status of drinking, snoring intensity, hypolipidemic drugs usage, hypoglycemic drugs usage. Analysis 1 excluded participants experienced hypertension during the first 2 years of follow-up. Analysis 2 excluded participants experienced diabetes during follow-up. Analysis 3 excluded participants experienced myocardial infarction and stroke during follow-up. Analysis 4 excluded all-cause death during follow-up.

### 3.3 Subgroup Analysis

In sub-study 1, the findings indicated that the associations were more pronounced in individuals aged less than 60 years (HR: 1.372, 95% CI: 1.252–1.504) than in individuals aged 60 years and older (HR: 0.988, 95% CI: 0.771–1.266) (*p *for interaction < 0.001) (Table 7). In sub-study 2, the findings indicated that the associations were more pronounced in individuals aged 60 years and older (HR: 1.258, 95% CI: 1.063–1.488) than in individuals aged less than 60 years (HR: 1.109, 95% CI: 1.053–1.169) (*p *for interaction < 0.001), in women (HR: 1.245, 95% CI: 1.135–1.366) than in men (HR: 1.051, 95% CI: 0.990–1.115) (*p *for interaction < 0.001), in individuals with BMI less than 28 (HR: 1.176, 95% CI: 1.116–1.239) than individuals with BMI of 28 and above (HR: 0.999, 95% CI: 0.831–1.203) (*p *for interaction < 0.001) (Table 8).

**Table 7.** **Hazard ratios for risk of hypertension in different subgroups**.

Subgroup			Total number	Case number	*p* value	HR	95% CI	*p* for interaction
Age								<0.001
	≥60							
		Q1	2273	162	-	1.000	-	
		Q2	2285	176	0.781	0.969	0.773–1.213	
		Q3	2397	187	0.261	0.875	0.693–1.105	
		Q4	2267	206	0.923	0.988	0.771–1.266	
	<60							
		Q1	11,940	837	-	1.000	-	
		Q2	11,930	1150	<0.001	1.174	1.071–1.287	
		Q3	11,816	1382	<0.001	1.268	1.158–1.388	
		Q4	11,947	1725	<0.001	1.372	1.252–1.504	
Sex								0.250
	Men							
		Q1	9665	807	-	1.000	-	
		Q2	10,582	1072	0.038	1.105	1.006–1.215	
		Q3	11,094	1294	0.002	1.163	1.060–1.277	
		Q4	11,749	1640	<0.001	1.290	1.175–1.417	
	Women							
		Q1	4548	192	-	1.000	-	
		Q2	3633	254	0.008	1.302	1.070–1.584	
		Q3	3119	275	<0.001	1.407	1.152–1.719	
		Q4	2465	291	<0.001	1.501	1.212–1.859	
BMI								0.051
	≥28							
		Q1	741	89	-	1.000	-	
		Q2	1351	170	0.841	1.027	0.788–1.339	
		Q3	2087	314	0.313	1.136	0.887–1.454	
		Q4	3183	562	0.030	1.304	1.027–1.656	
	<28							
		Q1	13,472	910	-	1.000	-	
		Q2	12,864	1156	0.001	1.159	1.060–1.268	
		Q3	12,126	1255	<0.001	1.214	1.109–1.330	
		Q4	11,031	1369	<0.001	1.329	1.211–1.460	

Model was adjusted for age, sex, body mass index, systolic blood pressure, diastolic blood pressure, total cholesterol, high-density lipoprotein cholesterol, low-density lipoprotein cholesterol, high sensitivity C-reactive protein, diabetes history, physical activity, status of smoking, status of drinking, snoring intensity, hypolipidemic drugs usage, hypoglycemic drugs usage.

**Table 8.** **Hazard ratios for risk of prehypertension in different subgroups**.

Subgroup			Total number	Case number	*p* value	HR	95% CI	*p* for interaction
Age								<0.001
	≥60							
		Q1	612	372	-	1.000	-	
		Q2	653	436	0.166	1.113	0.957–1.295	
		Q3	704	463	0.155	1.119	0.958–1.307	
		Q4	779	477	0.008	1.258	1.063–1.488	
	<60							
		Q1	4656	3637	-	1.000	-	
		Q2	4618	3846	<0.001	1.091	1.041–1.144	
		Q3	4566	3876	<0.001	1.118	1.065–1.174	
		Q4	4490	3875	<0.001	1.109	1.053–1.169	
Sex								<0.001
	Men							
		Q1	3062	2457	-	1.000	-	
		Q2	3389	2814	0.138	1.044	0.986–1.104	
		Q3	3751	3131	0.029	1.065	1.006–1.127	
		Q4	4064	3329	0.106	1.051	0.990–1.115	
	Women							
		Q1	2206	1552	-	1.000	-	
		Q2	1882	1468	<0.001	1.142	1.060–1.230	
		Q3	1519	1208	<0.001	1.161	1.071–1.259	
		Q4	1205	1023	<0.001	1.245	1.135–1.366	
BMI								<0.001
	≥28							
		Q1	192	168	-	1.000	-	
		Q2	308	265	0.658	0.955	0.780–1.170	
		Q3	482	415	0.801	1.025	0.847–1.239	
		Q4	848	714	0.995	0.999	0.831–1.203	
	<28							
		Q1	5076	3841	-	1.000	-	
		Q2	4963	4017	<0.001	1.112	1.062–1.165	
		Q3	4788	3924	<0.001	1.149	1.095–1.205	
		Q4	4421	3638	<0.001	1.176	1.116–1.239	

Model was adjusted for age, sex, body mass index, systolic blood pressure, diastolic blood pressure, total cholesterol, high-density lipoprotein cholesterol, low-density lipoprotein cholesterol, high sensitivity C-reactive protein, diabetes history, physical activity, status of smoking, status of drinking, snoring intensity, hypolipidemic drugs usage, hypoglycemic drugs usage.

## 4. Discussion

In this research, we examined the associations between the TyG index and the risk of hypertension and prehypertension in a Chinese population of North China. In this study, we demonstrated that the risk of hypertension and prehypertension exhibited a rising trend corresponding to higher quartiles of the TyG index. In addition, our results revealed a linear relationship between the TyG index and the risk of new-onset hypertension, and a non-linear relationship between the TyG index and the risk of new-onset prehypertension.

Consistent with our results, the findings of recent studies revealed that elevated levels of the TyG index were linked to a heightened risk of developing hypertension [9,10,11]. Furthermore, two studies investigated the relationship between the TyG index and the risk of hypertension in individuals aged 45 years and older [12] and in non-overweight healthy individuals [13]. In a separate investigation, Ishida et al. [14] examined the correlation between the TyG index and the risk of hypertension within a general Japanese population. The annual incidence of hypertension increased in parallel with the TyG index. Sawaf et al. [15] analyzed data from 10,937 non-diabetic individuals in the National Health and Nutrition Examination Survey, and found that patients with a TyG index higher than 8.96 had an odds ratio of 1.75–3.93 for hypertension compared with those with a TyG index below than 8.11.

Furthermore, several studies have examined the association between the TyG index and the risk of prehypertension. Xie et al. [16] conducted a cross-sectional study in the Chinese population and found that the risk of prehypertension increased across quartiles of the TyG index. In another study, Zhang et al. [17] reported a significant association between the TyG index and both prehypertension and hypertension. Wang et al. [18] found that individuals situated in the highest quartile of the TyG index exhibited a heightened risk of both prehypertension and hypertension when compared to those in the lowest quartile. Additionally, Lee et al. [19] examined data from the Korea National Health and Nutrition Examination Survey and found that individuals in the highest quartile of the TyG index had substantially higher risks of elevated blood pressure, prehypertension, and hypertension than those in the lowest quartile. Lastly, Xu et al. [20] demonstrated a significant correlation between the TyG index and the risk of prehypertension among Japanese individuals with normoglycemia.

Furthermore, our study indicated that the association between the TyG index and hypertension risk was more pronounced in individuals aged less than 60 years than in those aged 60 years and older, and in women than in men. In addition, the associations of the TyG index with risk and prehypertension were stronger in individuals aged 60 years and older than in those aged less than 60 years, in women than in men, and in individuals with a BMI of less than 28 than in those with a BMI of 28 and above. In this regard, Lee et al. [21] analyzed data from 5414 participants in the Korean Genome and Epidemiology Study aged 40 to 69 years. TyG and TyG-BMI were associated with new-onset hypertension only in women. Sun et al. [22] conducted a retrospective cohort study of 3465 Chinese individuals who participated in annual health check-ups. The incidence of hypertension increased across the quartiles of the TyG index for both genders. Nevertheless, after controlling for other variables, the highest quartile of the TyG index was found to be linked to an increased risk of hypertension solely in women. Wang et al. [10] performed a retrospective cohort study involving 3413 eligible participants drawn from the China Health and Nutrition Survey. Their findings suggested that, in comparison to participants with a TyG index of ≤ 8.41, those with a higher TyG index appeared to have increased odds of developing hypertension. Furthermore, this relationship was more pronounced among participants aged ≤ 65 years and among women. In another study, Yang et al. [23] conducted a meta-analysis of 35,848 participants from 7 cohort studies. The highest category of the TyG index demonstrated a greater risk of hypertension compared to the lowest category. Nevertheless, subgroup analysis indicated that the association between the TyG index and hypertension was not affected by age, sex, BMI, participant ethnicity, or follow-up duration.

Furthermore, some studies have explored the relationship between the TyG index and the risk of different types of hypertension. Cai et al. [24] revealed that a higher TyG index correlates with an elevated risk of isolated diastolic hypertension (IDH) and systolic-diastolic hypertension (SDH). Nevertheless, no substantial connection was found between the TyG index and isolated systolic hypertension (ISH). In another investigation, Shan et al. [25] analyzed data from the China Health and Retirement Longitudinal Study. Their findings indicated that individuals in the highest quartile of the TyG index had a higher risk of stage 1 hypertension, stage 2 hypertension, ISH, IDH, and SDH compared with those in the lowest quartile. Emlek et al. [26] demonstrated that the TyG index serves as an independent predictor of non-dipper hypertension. Additionally, Yang et al. [27] confirmed a significant correlation between the TyG index and the risk of resistant hypertension.

Obesity-related chronic inflammation plays a crucial role in the development of IR and the metabolic syndrome. Pro-inflammatory cytokines can induce IR in adipose tissue, skeletal muscle, and the liver by disrupting insulin signaling. Chronic inflammation may be linked to localized IR through autocrine/paracrine cytokine signaling and systemic IR via endocrine cytokine signaling, all of which play important roles in the abnormal metabolic condition [28]. Hypertension is associated with disrupted metabolic homeostasis and can be considered a metabolic disorder [29,30]. Research has indicated a correlation between IR, hyperinsulinemia, central/visceral obesity, and hypertension. Previous studies have highlighted that untreated hypertensive patients exhibit compensatory hyperinsulinemia, show resistance to insulin-mediated glucose uptake, and often present with concurrent lipid abnormalities [31]. Therefore, it is determined that all hemodynamic and metabolic disorders associated with essential hypertension and IR are intricately connected. The clinical strategy for managing a patient exhibiting any of these abnormalities should encompass the entire cluster, with treatment focused on improving the overall hemodynamic and metabolic profile.

## 5. Limitations

Several limitations must be recognized. Firstly, the hyperinsulinemic-euglycemic clamp (HEC) is considered the gold standard for assessing insulin sensitivity [32]. Nevertheless, HEC data were not available in the Kailuan study. Secondly, since the data for this research were derived from a predominantly male Chinese cohort, it is unclear whether the results can be generalized to other ethnic groups. Lastly, although the model was adjusted for various covariates, some potential confounding factors, such as family history of cardiovascular disease, were not accounted for. Further cohort studies are needed to confirm these findings and examine the impact of TyG index on the risk of hypertension and prehypertension.

## 6. Conclusions

An elevated TyG index is associated with increased risk of hypertension and prehypertension. The assessment of the TyG index may serve as a screening tool to recognize individuals who are at an increased risk of hypertension and prehypertension.

## Data Availability

The data that support the findings of this study are available from the corresponding author upon reasonable request.
